# Association of Blood Alcohol and Alcohol Use Disorders with Emergency Department Disposition of Trauma Patients

**DOI:** 10.5811/westjem.2021.9.51376

**Published:** 2022-02-28

**Authors:** Wirachin Hoonpongsimanont, Ghadi Ghanem, Preet Sahota, Abdullah Arif, Cristobal Barrios, Soheil Saadat, Shahram Lotfipour

**Affiliations:** * University of California, Irvine School of Medicine, Department of Emergency Medicine, Orange, California; † Eisenhower Medical Center, Department of Emergency Medicine, Rancho Mirage, California; ‡ University of California, Irvine School of Medicine, Department of Surgery, Division of Trauma, Burns, and Critical Care & Acute Care Surgery, Orange, California

## Abstract

**Introduction:**

Trauma patients who present to the emergency department (ED) intoxicated or with an alcohol use disorder (AUD) undergo more procedures and have an increased risk of developing complications. However, how AUD and blood alcohol concentration (BAC) impact a trauma patient’s disposition from the ED remains inconclusive. In this study we aimed to identify the associations between positive BAC or an AUD with admission to the hospital, including the intensive care unit (ICU).

**Methods:**

This was a retrospective study analyzing data from 2010–2018 at a university-based, Level I trauma ED. Included in the study were 4,699 adult trauma patients who completed the Alcohol Use Disorders Identification Test (AUDIT) and had blood alcohol content test results.

**Results:**

Positive BAC was associated with hospital admission and ICU admission after adjusting for injury severity score (ISS) (odds ratio 1.5 and 1.3, respectively). The AUDIT was only correlated with hospital and ICU admission in patients with ISS of 1 to 15. By increasing risk of AUD (low, moderate, high, and likely alcohol dependent) the proportion of ICU admissions rose from 29.3% to 37.3%, 40.0% and 42.0% (P <0.01). The results did not change significantly by adjustment for the age of patients.

**Conclusion:**

BAC is associated with increasing ED disposition to the hospital or ICU. Furthermore, self-reported alcohol use was associated with an increased risk of hospital or ICU admission in patients with minor or moderate injuries. Further studies to determine viable options to decrease admission rates in these patients are warranted.

## INTRODUCTION

Alcohol consumption can play a significant role in trauma patients’ visits to the emergency department (ED). Previous studies have found that between 33.5% and 47% of trauma patients presenting to the ED have a positive blood alcohol concentration (BAC).^1^, ^2^ Furthermore, a systematic review of trauma centers across the United States found that 26.2–62.5% of visits to trauma centers were alcohol related.^3^

Alcohol consumption prior to injury has been shown to lead to higher rates of infection-related complications and more diagnostic tests, which ultimately results in longer lengths of stay, extra procedures, higher hospitalization costs, and a higher probability of hospital admission.^4^–^6^ Patients with positive BACs are most likely to present to the ED between midnight and 2 am, have a higher injury severity score (ISS), and have an increased risk of head trauma.^6^–^8^ While BAC is the most commonly used tool for assessing patients’ acute alcohol consumption levels, it cannot capture and often misses habitual consumption levels.^9^,^10^ Blood alcohol concentration has also been shown to miss more than one third of trauma patients with a current alcohol use disorder (AUD).^11^ For that reason, many EDs have incorporated increased screening for AUDs in their patient populations.

Alcohol use disorder has become increasingly prevalent in trauma patients. A study conducted across four major trauma centers in Los Angeles found that 24.0% of patients were characterized with AUD.^12^ Furthermore, on a national level, 34.3% of adult ED patients have either risky drinking, problem drinking, or alcohol-dependence behaviors.^13^ Researchers have also found upward trends in alcohol-related ED visits over a nine-year period, indicating an increase in problematic drinking in this population over time.^14^ In the trauma population, AUD and substance use disorders in general are associated with increased mortality.^15^ Other studies beyond emergency medicine have found that in patients who undergo surgery, having an AUD was strongly correlated with hospital readmission. The readmitted patients with an AUD also had increased length of stay, hospitalization costs, and risk of death.^16^

Due to the increasing prevalence of AUD in trauma patients, the American College of Surgeons Committee on Trauma has mandated that Level I trauma centers provide alcohol screening to all trauma patients and interventions for those with high risk of AUD. The current standard alcohol screening tool is the Alcohol Use Disorders Identification Test (AUDIT). The AUDIT is a self-reported, 10-item questionnaire developed by the World Health Organization; it is designed to assess patients’ alcohol consumption habits over the prior year.^17^ Upon completion of the questionnaire, a score is generated, which ranges from 0–40 and categorizes the patient as either low risk for alcohol dependence (0–7) or high risk for alcohol dependence (8–19), or likely alcohol dependent (20–40). Patients with AUDIT scores of 8–19 are offered a personalized brief intervention aimed at reducing their alcohol consumption, which has proven effective.^18^–^20^ Despite the self-report bias present in AUDIT, the survey has been shown to be a reliable and well-validated measure to assess habitual alcohol intake in patients.^21^–^25^ Delivering the AUDIT test through a self-administered, computerized system has also been shown to be feasible and may reduce biases associated with alcohol reporting.^22^,^26^

While many studies have found correlations between alcohol consumption and a myriad of other variables, the evidence for associations between AUDIT and disposition in trauma patients is limited. In this study, we aimed to identify the correlation between acute and chronic alcohol consumption, as defined by BAC and AUDIT, respectively, with disposition of trauma patients from the ED.

## METHODS

### Study Setting and Design

We conducted a retrospective, chart review study on databases that were obtained at a Level I trauma center, university-based ED between 2010–2018. Patients were included if they were over 18 years of age and met trauma activation criteria (Supplemental Document). All these patients completed the AUDIT in either Spanish or English. The study was reviewed and approved by the university’s institutional review board as an exempt category. Patient informed consent was not applicable to this study.

**Population Health Research Capsule**

What do we already know about this issue?

*Alcohol consumption has a significant impact on trauma patients’ care in the emergency department.*

What was the research question?

*What is the association between alcohol consumption and disposition of trauma patients from the ED?*

What was the major finding of the study?

*Positive blood alcohol concentration and self-reported alcohol use were associated with hospital and intensive care unit admission in trauma patients.*

How does this improve population health?

*Effective alcohol screening and intervention could help reduce the admission rate in trauma patients.*

### Study Protocol

We obtained our data from two databases: the hospital Trauma Registry and the Computerized Alcohol Screening and Intervention (CASI) program database. The Trauma Registry database compiles patient information from all trauma patients as part of quality assurance. Data analysts obtained patient demographics; nurse abstractors obtained information on patient injuries, treatments, BAC, and diagnoses/outcomes. We obtained ED disposition (death, intensive care unit [ICU] admission, hospital admission, and discharge from the ED), BAC, and ISS^27^ from this database. When patients were first admitted to the ED, and trauma surgeons deemed it appropriate, the patients received venous blood draws to measure BAC as part of evaluation protocols. We included only patients who had BAC measurement results.

The CASI database was compiled by trained research associates (RA) who administered the AUDIT to trauma patients. Implementation of AUDIT screening was standard of care for trauma patients from 8 am to midnight in the ED, and 8 am to noon in the inpatient units. All trauma patients were approached to complete the AUDIT when they were clinically stable during their stay in the ED or inpatient units. Patients completed the AUDIT on a CASI tablet privately, unless a patient specifically requested assistance from an RA. Responses to individual questions were kept confidential. The AUDIT score is shared with the patient, and a printout of the score is attached to the patient’s medical record. We excluded patients who were on a psychiatric hold, incarcerated, or pregnant. For those with cognitive impairments such as acute intoxication, altered mental status, and critical illness, the RAs approached the patients once their conditions were resolved. The AUDIT results and demographic information were electronically recorded and automatically stored in a secure hospital database. We extracted patient demographic data and AUDIT scores from this database.

The two databases were linked by a unique identifier for each patient using Python Language Reference version 2.7 (Python Software Foundation, Wilmington, DE).

### Statistical Analysis

Frequencies are reported as N (%). We studied the distribution of categorical variables using the chi-square, or chi-square for trend, statistical test. Associations of BAC and AUDIT with ICU admission and hospital admission were studied by calculating odds ratios (OR) in each level of ISS. We examined the homogeneity of estimated OR among levels of ISS using the Breslow-Day statistical test, and if the homogeneity was not rejected we reported a Mantel-Haenszel common OR. Statistical analyses were performed using SPSS Statistics 25 for Windows (IBM Corporation, Armonk, NY).

## RESULTS

We identified a total of 4,699 adult trauma patients with known BAC who had completed the AUDIT questionnaire. Of these patients 3116 were male and 1583 were female (Table 1). The mean age of female patients was 51.4 years (±30.20) compared to 42.2 years (±19.86) in male patients (*P* <0.001). While male patients were younger, a higher percentage of them presented with an ISS score greater than 15 (*P* = 0.001). A total of 3551 (75.6%) patients had a BAC of zero upon arrival to the ED; 243 (5.2%) patients presented with a BAC greater than 250 milligrams per deciliter (mg/dL). A greater percentage of male patients presented with positive BAC (Table 1) as compared to female patients (*P* < 0.001). A similar pattern was observed with AUDIT scores.

### Associated Factors with Hospital and ICU Admission: All Patients

Our results showed an association between positive blood alcohol with both hospital admission and ICU admission (among those who had been admitted to the hospital) after adjusting for ISS (Mantel-Haenszel OR: 1.5 [1.2 – 1.9] and 1.3 [1.1 – 1.5], respectively) (Table 2). The association was still significant after adjusting for age groups (18–30, 31–50, 51+) and ISS (Mantel-Haenszel OR: 1.7 [1.4–2.1] and 1.4 [1.2–1.7], respectively (Appendices 1 and 2). However, we did not find a statistically significant association between AUDIT score when considered as a score of zero (ie, self-reported abstainer) vs “1 or more” scores, and hospital (*P* = 0.763) or ICU admission (*P* = 0.494) after adjustment for ISS.

### Associated Factors with Hospital Admission in Patients with Injury Severity Score 1–15

**Figure 1:**
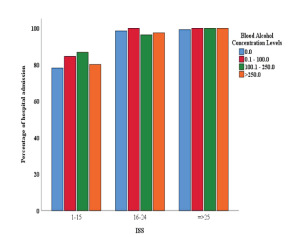
Association of hospital admission with blood alcohol concentration, per Injury Severity Score category. *ISS*, Injury Severity Score.

There was a statistically significant association between hospital admission and BAC among patients with ISS of 1–15 (Figure 1). By increasing BAC (from 0 mg/dL to 0.1–100 mg/dL, 100.1–250 mg/dL, and >250 mg/dL) the proportion of hospital admission rose from 78.1% to 84.5%, 86.8% and 80.1%, respectively (*P* = 0.001). The association remains statistically significant (*P* < 0.001) after adjustment for age (Appendix 3).

**Figure 2:**
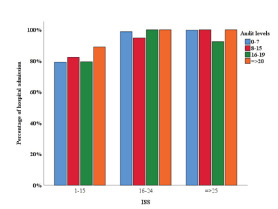
Association of hospital admission with AUDIT* scores, per Injury Severity Score category. *AUDIT, Alcohol Use Disorders Identification Test; *ISS*, Injury Severity Score.

A similar association was observed between hospital admission and AUDIT scores in patients with an ISS of 1–15 (Figure 2). By increasing AUDIT levels (from 0–7 to 8–15, 16–19, and ≥20) the proportion of hospital admissions rose from 79.1% to 82.2%, 79.4% and 88.9%, respectively (P = 0.016). The association remained statistically significant (*P* < 0.001) after adjustment for age (Appendix 4).

### Associated Factors with ICU Admission in Patients with ISS 1–15 Who Were Amitted to the Hospital

**Figure 3:**
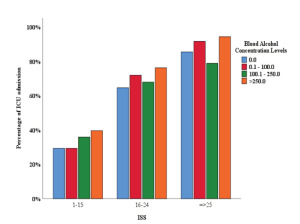
Association of ICU admission with blood alcohol concentration, per Injury Severity Score category. *ISS*, Injury Severity Score.

**Figure 4:**
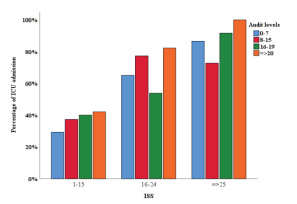
Association of intensive care unit admission with AUDIT* scores, per Injury Severity Score category. *AUDIT, Alcohol Use Disorder Identification test; *ISS*, Injury Severity Score.

Among the patients who had been admitted into the hospital, we found a statistically significant association between ICU admission and BAC in patients with ISS of 1–15 (Figure 3). The proportion of ICU admissions in patients with BAC up to 100 mg/dL was 29.3%. The proportion of ICU admissions rose to 35.9% and 39.6% by increasing BAC to 100.1–250 mg/dL and >250 mg/dL, respectively (*P* = 0.001). The association remained statistically significant (*P* <0.001) after adjustment for age (Appendix 5). We observed a similar association between ICU admission and AUDIT scores in the same group of patients (Figure 4). By increasing AUDIT levels (from 0–7 to 8–15, 16–19, and ≥ 20) the proportion of ICU admissions rose from 29.3% to 37.3%, 40.0% and 42.0%, respectively. (*P* <0.001). The association remained statistically significant (*P* <0.001) after adjustment for age (Appendix 6).

## DISCUSSION

When we considered BAC as a dichotomous variable (BAC 0 mg/dL and BAC >0 mg/dL), we found that positive blood alcohol is associated with increased risk of hospital and ICU admission. Having a positive BAC may complicate the initial patient presentation and subsequent tests, especially in patients with lower ISS, whereas the effects of chronic alcohol use may not have yet manifested. Management and differentials of patients are also complicated by alcohol use, as intoxication may mask the effects of a stroke or head injury.^28^ Physicians may, therefore, opt to admit these patients until they are stable and can be properly assessed. Furthermore, patients who are undomiciled are more than twice as likely to present with positive BAC after a trauma than a comparative sample of domiciled patients.^29^ This may complicate the discharge process for lower ISS patients who do not have reliable transportation, a shelter to return to, or a follow-up plan. This population also has a higher rate of psychiatric-related admissions.^29^

Intoxicated trauma patients are less likely to sustain severe injuries.^30^,^31^ However, they are more likely to present with a depressed Glasgow Coma Scale score,^32^ which is usually associated with intracranial trauma, hypoxia, or shock from associated injuries. This may prompt physicians to admit these patients for further investigation and work-up. Furthermore, these patients may be cognitively impaired, even as their blood alcohol levels approach zero or may go on to develop complications from alcohol withdrawal syndrome that will eventually require hospital admission.^33^

Trauma patients who are BAC positive are more likely to have a pre-existing condition of chronic alcohol use, cirrhosis, coagulopathy, chronic pulmonary condition, chronic obstructive pulmonary disease, or chronic drug use.^34^ Another study found that 66% of frequent binge drinkers and 10% of infrequent binge drinkers were found to be BAC positive upon admission.^35^ Binge drinking is known to dysregulate adipocyte and liver function, thereby contributing to metabolic derangement and alcoholic liver disease.^36^ A single binge-alcohol session can also modulate immune system functioning.^37^ The combination of pre-existing conditions and impaired immune function may, therefore, contribute to the increased risk of infections observed in patients with positive BAC.^38^ Other studies have indicated that patients with positive BAC also have a higher risk of developing pneumonia.^39^ These complications may encourage a physician to admit patients with positive BAC either due to incidental findings (not related to the trauma) or fear of patient deterioration.

When we categorized AUDIT into four levels (0–7, 8–15, 16–19, ≥20) we observed statistically significant associations between AUDIT levels and both ICU and hospital admission only in patients with ISS of 1–15. The AUDIT is a reflection of the patient’s perceived long-term alcohol consumption habits. Chronic alcohol use has been found to contribute to a plethora of diseases and immune dysfunctions, as well as comorbidities with other psychological disorders.^40^,^41^ In severely injured patients (ISS greater than 15), the health consequences of chronic alcohol use was likely to have been masked or superseded by the traumatic injury. But when the injuries were minor (ISS 1–15), the effects of chronic alcohol use on the patient’s health became more prominent and possibly contributed to hospital admission. Previous studies have identified that orthopedic trauma patients with a history of AUD are more likely to be admitted and have an increased length of stay.^42^,^43^ However, many of these studies were unable to discern a cause behind these statistics. Our results would indicate that the skew in data may have been primarily due to patients with minor injuries but higher risk of AUD. We therefore recommend that future studies designed to discern the effects of AUD on patient outcomes should further stratify this population by ISS.

## LIMITATIONS

Patients with prolonged altered mental status due to various reasons, including intoxication and being intubated, completed the AUDIT at a later stage of their hospital stay or were excluded from our study. Although most patients completed the study within 48 hours while they were still in the ED, the accuracy of AUDIT responses might diminish if the patients completed the survey near the time of their discharge. Trauma patients with the inability to personally complete the survey due to their injuries completed the AUDIT with assistance from research personnel, which may have introduced social desirability response biases in these patients. Furthermore, ethanol tolerance may skew the symptoms of intoxication for patients with a history of AUD, thereby complicating the scale of BAC intoxication and clinical intoxication.^44^ Systemic biases have also been found in determining which patients are tested for BAC, which may have caused us to miss some patients on initial presentation.^45^

Other patient care outcomes besides hospital admission, such as alcohol withdrawal symptoms and poor surgical outcomes, may impact patient management but were not available in our databases.

## CONCLUSION

Blood alcohol concentration is a reflection of acute alcohol use, often correlated with binge drinking and adverse effects on human health. The presence of BAC was found to be associated with hospital and ICU admissions after adjustment for Injury Severity Score; therefore, screening BAC might expedite disposition of trauma patients in the ED. The Alcohol Use Disorder Identification Test is a self-reported reflection of perceived alcohol consumption habits and possible chronic alcohol use. Our study found that AUDIT is associated with an increased risk of hospital or ICU admission in minor or moderately injured trauma patients only.

## Supplementary Information







## Figures and Tables

**Table 1:** Demographics of adult trauma patients included in the study.

	Gender		Total

	Male	Female	
Age (Mean ± SD)	42.2 ± 19.86	51.4 ± 30.20	45.3 ± 24.24
ISS			
1–15	2,394 (76.8%)	1,292 (81.6%)	3,686 (78.4%)
16–24	460 (17.8%)	177 (11.2%)	637 (13.6%)
=>25	262 (8.4%)	114 (7.2%)	376 (8.0%)
Blood Alcohol Concentration (mg/dL)			
0.0	2,205 (70.8%)	1,346 (85.0%)	3,551 (75.6%)
0.1 – 100.0	318 (10.2%)	78 (4.9%)	396 (8.4%)
100.1 – 250.0	398 (12.8%)	111 (7.0%)	509 (10.8%)
>250.0	195 (6.3%)	48 (3.0%)	243 (5.2%)
AUDIT score			
0–7	2,582 (82.9%)	1,494 (94.4%)	4,076 (86.7%)
8–15	347 (11.1%)	63 (4.0%)	410 (8.7%)
16–19	79 (2.5%)	10 (0.6%)	89 (1.9%)
=>20	108 (3.5%)	16 (1.0%)	124 (2.6%)
ED disposition			
Discharged	470 (15.1%)	291 (18.4%)	761 (16.2%)
In Hospital (non-ICU)	1,482 (47.7%)	816 (51.6%)	2,298 (49.0%)
ICU	1,157 (37.2%)	474 (30.0%)	1,631 (34.8%)
Dead	1 (0.0%)	1 (0.1%)	2 (0.0%)

*SD*, standard deviation; *ISS*, Injury Severity Score; *mg*, milligram; *dL*, deciliter; *AUDIT*, Alcohol Use Disorders Identification Test; *ED*, emergency department; *ICU*, intensive care unit.

**Table 2:** Association of blood alcohol with hospital admission and intensive care unit admission adjusted for Injury Severity Score.

ISS levels				Hospital admission				ICU admission			

				No	Yes*	Total	Odds ratio (95% CI)	No	Yes	Total	Odds ratio (95% CI)
1–15	BA	Negative	Count	614	2,194	2,808	1.53 (1.2 – 1.9)	1,547	641	2,188	1.3 (1.1 – 1.5)
			row %	21.9%	78.1%	100.0%		70.7%	29.3%	100.0%	
		Positive	Count	134	734	868		480	254	734	
			row %	15.4%	84.6%	100.0%		65.4%	34.6%	100.0%	
		Total	Count	748	2,928	3,676		2,027	895	2,922	
			row %	20.3%	79.7%	100.0%		69.4%	30.6%	100.0%	
16–24	BA	Negative	Count	7	479	486	0.7 (0.2 – 2.8)	169	309	478	1.4 (0.9 – 2.1)
			row %	1.4%	98.6%	100.0%		35.4%	64.6%	100.0%	
		Positive	Count	3	146	149		41	105	146	
			row %	2.0%	98.0%	100.0%		28.1%	71.9%	100.0%	
		Total	Count	10	625	635		210	414	624	
			row %	1.6%	98.4%	100.0%		33.7%	66.3%	100.0%	
=>25	BA	Negative	Count	2	255	257	1.0(1.0 – 1.0)	37	217	254	1.0 (0.6 – 2.0)
			row %	0.8%	99.2%	100.0%		14.6%	85.4%	100.0%	
		Positive	Count	0	115	115		16	98	114	
			row %	0.0%	100.0%	100.0%		14.0%	86.0%	100.0%	
		Total	Count	2	370	372		53	315	368	
			row %	0.5%	99.5%	100.0%		14.4%	85.6%	100.0%	
Total	BA	Negative	Count	623	2,928	3,551	Mantel-Haenszel common odds ratio:	1,753	1,167	2,920	Mantel-Haenszel common odds ratio:
			row %	17.5%	82.5%	100.0%		60.0%	40.0%	100.0%	
		Positive	Count	137	995	1,132		537	457	994	
			row %	12.1%	87.9%	100.0%	1.5 (1.2 – 1.9) *P* <0.001	54.0%	46.0%	100.0%	1.3 (1.1 – 1.5) *P* = 0.002
		Total	Count	760	3,923	4,683		2,290	1,624	3,914	
			row %	16.2%	83.8%	100.0%		58.5%	41.5%	100.0%	

* Including 2 dead.

*ISS*, Injury Severity Score; *CI*, confidence interval; *ICU*, intensive care unit; *BA*, blood alcohol.
