# Supplementary material for: Association of Blood Alcohol and Alcohol Use Disorders with Emergency Department Disposition of Trauma Patients
Source: West J Emerg Med. 2022 Feb 28;23(2):158–65. doi: 10.5811/westjem.2021.9.51376 (PMC8967454; doi:10.5811/westjem.2021.9.51376)
Supplement: Supplementary file 1 [file wjem-23-158-s001.docx]

**Appendix 1**. Mantel-Haenszel Common Odds Ratio Calculations for the Association Between Positive Blood Alcohol and Hospital Admission - with Adjustment for Age Group and Injury Severity Score (ISS).

**Crosstab**

Age groups (30 50)

Hospital Admission

Discharged

Admitted

Total

ISS levels

BAC 0

vs Any

18-30

1-15

0.00

Count

%

253

27.2%

676

72.8%

929

100.0%

1.00

Count

%

78

18.1%

353

81.9%

431

100.0%

Total

Count

%

331

24.3%

1029

75.7%

1360

100.0%

BAC 0

vs Any

16-24

0.00

Count

%

3

2.7%

110

97.3%

113

100.0%

1.00

Count

%

1

1.5%

65

98.5%

66

100.0%

Total

Count

%

4

2.2%

175

97.8%

179

100.0%

BAC 0

vs Any

=>25

0.00

Count

%

2

2.5%

79

97.5%

81

100.0%

1.00

Count

%

0

0.0%

58

100.0%

58

100.0%

Total

Count

%

2

1.4%

137

98.6%

139

100.0%

BAC 0

vs Any

Total

0.00

Count

%

258

23.0%

865

77.0%

1123

100.0%

1.00

Count

%

79

14.2%

476

85.8%

555

100.0%

Total

Count

%

337

20.1%

1341

79.9%

1678

100.0%

BAC 0

vs Any

31-50

1-15

0.00

Count

%

165

24.1%

521

75.9%

686

100.0%

1.00

Count

%

36

13.8%

224

86.2%

260

100.0%

Total

Count

%

201

21.2%

745

78.8%

946

100.0%

BAC 0

vs Any

16-24

0.00

Count

%

1

0.8%

130

99.2%

131

100.0%

1.00

Count

%

1

2.1%

46

97.9%

47

100.0%

Total

Count

%

2

1.1%

176

98.9%

178

100.0%

BAC 0

vs Any

=>25

0.00

Count

%

69

100.0%

69

100.0%

Page 1

**Crosstab**

Age groups (30 50)

Hospital Admission

Discharged

Admitted

Total

ISS levels

1.00

Count

%

34

100.0%

34

100.0%

Total

Count

%

103

100.0%

103

100.0%

BAC 0

vs Any

Total

0.00

Count

%

166

18.7%

720

81.3%

886

100.0%

1.00

Count

%

37

10.9%

304

89.1%

341

100.0%

Total

Count

%

203

16.5%

1024

83.5%

1227

100.0%

BAC 0

vs Any

51-100

1-15

0.00

Count

%

195

16.4%

993

83.6%

1188

100.0%

1.00

Count

%

20

11.3%

157

88.7%

177

100.0%

Total

Count

%

215

15.8%

1150

84.2%

1365

100.0%

BAC 0

vs Any

16-24

0.00

Count

%

3

1.2%

239

98.8%

242

100.0%

1.00

Count

%

1

2.8%

35

97.2%

36

100.0%

Total

Count

%

4

1.4%

274

98.6%

278

100.0%

BAC 0

vs Any

=>25

0.00

Count

%

107

100.0%

107

100.0%

1.00

Count

%

23

100.0%

23

100.0%

Total

Count

%

130

100.0%

130

100.0%

BAC 0

vs Any

Total

0.00

Count

%

198

12.9%

1339

87.1%

1537

100.0%

1.00

Count

%

21

8.9%

215

91.1%

236

100.0%

Total

Count

%

219

12.4%

1554

87.6%

1773

100.0%

BAC 0

vs Any

Total

1-15

0.00

Count

%

613

21.9%

2190

78.1%

2803

100.0%

1.00

Count

%

134

15.4%

734

84.6%

868

100.0%

Page 2

**Crosstab**

Age groups (30 50)

Hospital Admission

Discharged

Admitted

Total

ISS levels

Total

Count

%

747

20.3%

2924

79.7%

3671

100.0%

BAC 0

vs Any

16-24

0.00

Count

%

7

1.4%

479

98.6%

486

100.0%

1.00

Count

%

3

2.0%

146

98.0%

149

100.0%

Total

Count

%

10

1.6%

625

98.4%

635

100.0%

BAC 0

vs Any

=>25

0.00

Count

%

2

0.8%

255

99.2%

257

100.0%

1.00

Count

%

0

0.0%

115

100.0%

115

100.0%

Total

Count

%

2

0.5%

370

99.5%

372

100.0%

BAC 0

vs Any

Total

0.00

Count

%

622

17.5%

2924

82.5%

3546

100.0%

1.00

Count

%

137

12.1%

995

87.9%

1132

100.0%

Total

Count

%

759

16.2%

3919

83.8%

4678

100.0%

Page 3

**Odds Ratio**

Age groups (30 50)

95% Confidence Interval

Lower

Upper

Value

ISS levels

Odds Ratio for BAC 0 vs Any (0.00 / 1.00)

For cohort Admission or death = Discharged

For cohort Admission or death = Admitted/dead

N of Valid Cases

18-30

1-15

1.694

1.274

2.251

1.505

1.200

1.888

0.888

0.837

0.943

1360

Odds Ratio for BAC 0 vs Any (0.00 / 1.00)

For cohort Admission or death = Discharged

For cohort Admission or death = Admitted/dead

N of Valid Cases

16-24

1.773

0.181

17.398

1.752

0.186

16.504

0.988

0.947

1.032

179

For cohort Admission or death = Admitted/dead

N of Valid Cases

=>25

0.975

0.942

1.010

139

Odds Ratio for BAC 0 vs Any (0.00 / 1.00)

For cohort Admission or death = Discharged

For cohort Admission or death = Admitted/dead

N of Valid Cases

Total

1.797

1.364

2.368

1.614

1.282

2.033

0.898

0.857

0.941

1678

Odds Ratio for BAC 0 vs Any (0.00 / 1.00)

For cohort Admission or death = Discharged

For cohort Admission or death = Admitted/dead

N of Valid Cases

31-50

1-15

1.971

1.330

2.919

1.737

1.248

2.419

0.882

0.827

0.940

946

Odds Ratio for BAC 0 vs Any (0.00 / 1.00)

For cohort Admission or death = Discharged

For cohort Admission or death = Admitted/dead

N of Valid Cases

16-24

0.354

0.022

5.773

0.359

0.023

5.622

1.014

0.970

1.060

178

.a

Odds Ratio for BAC 0 vs Any (0.00 / 1.00)

=>25

Odds Ratio for BAC 0 vs Any (0.00 / 1.00)

For cohort Admission or death = Discharged

Total

1.894

1.295

2.772

1.727

1.237

2.411

Page 4

**Odds Ratio**

Age groups (30 50)

95% Confidence Interval

Lower

Upper

Value

ISS levels

For cohort Admission or death = Admitted/dead

N of Valid Cases

0.912

0.868

0.957

1227

Odds Ratio for BAC 0 vs Any (0.00 / 1.00)

For cohort Admission or death = Discharged

For cohort Admission or death = Admitted/dead

N of Valid Cases

51-100

1-15

1.542

0.944

2.516

1.453

0.943

2.238

0.942

0.889

0.999

1365

Odds Ratio for BAC 0 vs Any (0.00 / 1.00)

For cohort Admission or death = Discharged

For cohort Admission or death = Admitted/dead

N of Valid Cases

16-24

0.439

0.044

4.342

0.446

0.048

4.175

1.016

0.960

1.075

278

.a

Odds Ratio for BAC 0 vs Any (0.00 / 1.00)

=>25

Odds Ratio for BAC 0 vs Any (0.00 / 1.00)

For cohort Admission or death = Discharged

For cohort Admission or death = Admitted/dead

N of Valid Cases

Total

1.514

0.944

2.428

1.448

0.943

2.222

0.956

0.915

1.000

1773

Odds Ratio for BAC 0 vs Any (0.00 / 1.00)

For cohort Admission or death = Discharged

For cohort Admission or death = Admitted/dead

N of Valid Cases

Total

1-15

1.533

1.249

1.882

1.417

1.194

1.680

0.924

0.893

0.956

3671

Odds Ratio for BAC 0 vs Any (0.00 / 1.00)

For cohort Admission or death = Discharged

For cohort Admission or death = Admitted/dead

N of Valid Cases

16-24

0.711

0.182

2.785

0.715

0.187

2.732

1.006

0.981

1.032

635

For cohort Admission or death = Admitted/dead

N of Valid Cases

=>25

0.992

0.982

1.003

372

Page 5

**Odds Ratio**

Age groups (30 50)

95% Confidence Interval

Lower

Upper

Value

ISS levels

Odds Ratio for BAC 0 vs Any (0.00 / 1.00)

For cohort Admission or death = Discharged

For cohort Admission or death = Admitted/dead

N of Valid Cases

Total

1.545

1.267

1.884

1.449

1.220

1.722

0.938

0.914

0.963

4678

**Mantel-Haenszel Common Odds Ratio Estimate**

OR Ln(OR)

Standard Error of Ln(OR)

Asymptotic Significance (2-sided)

1.722

0.543

0.105

0.000

1.401

2.116

0.337

0.749

Asymptotic 95% Confidence Interval

Common Odds Ratio

Lower Bound Upper Bound Lower Bound

Upper Bound

Ln(Common Odds Ratio)

The Mantel-Haenszel common odds ratio estimate is asymptotically normally distributed under the common odds ratio of 1.000 assumption. So is the natural log of the estimate.

Page 6
