# Supplementary material for: Association of Blood Alcohol and Alcohol Use Disorders with Emergency Department Disposition of Trauma Patients
Source: West J Emerg Med. 2022 Feb 28;23(2):158–65. doi: 10.5811/westjem.2021.9.51376 (PMC8967454; doi:10.5811/westjem.2021.9.51376)
Supplement: Supplementary file 2 [file wjem-23-158-s002.docx]

**Appendix 2.** Mantel-Haenszel Common Odds Ratio Calculations for the Association Between Positive Blood Alcohol and Intensive Care Unit (ICU) Admission - with Adjustment for Age Group and Injury Severity Score (ISS).

**Crosstab**

Age groups (30 50)

ICU admission

No

Yes

Total

ISS levels

18-30

1-15

BAC 0 vs Any

0.00

Count

%

502

74.5%

172

25.5%

674

100.0%

1.00

Count

%

238

67.4%

115

32.6%

353

100.0%

Total

Count

%

740

72.1%

287

27.9%

1027

100.0%

16-24

BAC 0 vs Any

0.00

Count

%

49

44.5%

61

55.5%

110

100.0%

1.00

Count

%

21

32.3%

44

67.7%

65

100.0%

Total

Count

%

70

40.0%

105

60.0%

175

100.0%

=>25

BAC 0 vs Any

0.00

Count

%

14

17.7%

65

82.3%

79

100.0%

1.00

Count

%

10

17.2%

48

82.8%

58

100.0%

Total

Count

%

24

17.5%

113

82.5%

137

100.0%

Total

BAC 0 vs Any

0.00

Count

%

565

65.5%

298

34.5%

863

100.0%

1.00

Count

%

269

56.5%

207

43.5%

476

100.0%

Total

Count

%

834

62.3%

505

37.7%

1339

100.0%

31-50

1-15

BAC 0 vs Any

0.00

Count

%

385

74.3%

133

25.7%

518

100.0%

1.00

Count

%

138

61.6%

86

38.4%

224

100.0%

Total

Count

%

523

70.5%

219

29.5%

742

100.0%

16-24

BAC 0 vs Any

0.00

Count

%

55

42.6%

74

57.4%

129

100.0%

1.00

Count

%

11

23.9%

35

76.1%

46

100.0%

Total

Count

%

66

37.7%

109

62.3%

175

100.0%

=>25

BAC 0 vs Any

0.00

Count

%

10

14.7%

58

85.3%

68

100.0%

Page 1

**Crosstab**

Age groups (30 50)

ICU admission

No

Yes

Total

ISS levels

1.00

Count

%

3

9.1%

30

90.9%

33

100.0%

Total

Count

%

13

12.9%

88

87.1%

101

100.0%

Total

BAC 0 vs Any

0.00

Count

%

450

62.9%

265

37.1%

715

100.0%

1.00

Count

%

152

50.2%

151

49.8%

303

100.0%

Total

Count

%

602

59.1%

416

40.9%

1018

100.0%

51-100

1-15

BAC 0 vs Any

0.00

Count

%

657

66.2%

335

33.8%

992

100.0%

1.00

Count

%

104

66.2%

53

33.8%

157

100.0%

Total

Count

%

761

66.2%

388

33.8%

1149

100.0%

16-24

BAC 0 vs Any

0.00

Count

%

65

27.2%

174

72.8%

239

100.0%

1.00

Count

%

9

25.7%

26

74.3%

35

100.0%

Total

Count

%

74

27.0%

200

73.0%

274

100.0%

=>25

BAC 0 vs Any

0.00

Count

%

13

12.1%

94

87.9%

107

100.0%

1.00

Count

%

3

13.0%

20

87.0%

23

100.0%

Total

Count

%

16

12.3%

114

87.7%

130

100.0%

Total

BAC 0 vs Any

0.00

Count

%

735

54.9%

603

45.1%

1338

100.0%

1.00

Count

%

116

54.0%

99

46.0%

215

100.0%

Total

Count

%

851

54.8%

702

45.2%

1553

100.0%

Total

1-15

BAC 0 vs Any

0.00

Count

%

1544

70.7%

640

29.3%

2184

100.0%

1.00

Count

%

480

65.4%

254

34.6%

734

100.0%

Page 2

**Crosstab**

Age groups (30 50)

ICU admission

No

Yes

Total

ISS levels

Total

Count

%

2024

69.4%

894

30.6%

2918

100.0%

16-24

BAC 0 vs Any

0.00

Count

%

169

35.4%

309

64.6%

478

100.0%

1.00

Count

%

41

28.1%

105

71.9%

146

100.0%

Total

Count

%

210

33.7%

414

66.3%

624

100.0%

=>25

BAC 0 vs Any

0.00

Count

%

37

14.6%

217

85.4%

254

100.0%

1.00

Count

%

16

14.0%

98

86.0%

114

100.0%

Total

Count

%

53

14.4%

315

85.6%

368

100.0%

Total

BAC 0 vs Any

0.00

Count

%

1750

60.0%

1166

40.0%

2916

100.0%

1.00

Count

%

537

54.0%

457

46.0%

994

100.0%

Total

Count

%

2287

58.5%

1623

41.5%

3910

100.0%

Page 3

**Odds Ratio**

95% Confidence Interval

Age groups (30 50)

Lower

Upper

ISS levels

Value

Odds Ratio for BAC 0 vs Any (0.00 / 1.00)

For cohort ICU admission = 0.00

For cohort ICU admission = ICU admission

N of Valid Cases

18-30

1-15

1.410

1.064

1.870

1.105

1.015

1.203

0.783

0.643

0.955

1027

Odds Ratio for BAC 0 vs Any (0.00 / 1.00)

For cohort ICU admission = 0.00

For cohort ICU admission = ICU admission

N of Valid Cases

16-24

1.683

0.886

3.197

1.379

0.916

2.076

0.819

0.646

1.039

175

Odds Ratio for BAC 0 vs Any (0.00 / 1.00)

For cohort ICU admission = 0.00

For cohort ICU admission = ICU admission

N of Valid Cases

=>25

1.034

0.423

2.525

1.028

0.492

2.149

0.994

0.851

1.162

137

Odds Ratio for BAC 0 vs Any (0.00 / 1.00)

For cohort ICU admission = 0.00

For cohort ICU admission = ICU admission

N of Valid Cases

Total

1.459

1.160

1.835

1.158

1.056

1.271

0.794

0.692

0.911

1339

Odds Ratio for BAC 0 vs Any (0.00 / 1.00)

For cohort ICU admission = 0.00

For cohort ICU admission = ICU admission

N of Valid Cases

31-50

1-15

1.804

1.292

2.519

1.206

1.075

1.354

0.669

0.536

0.834

742

Odds Ratio for BAC 0 vs Any (0.00 / 1.00)

For cohort ICU admission = 0.00

For cohort ICU admission = ICU admission

N of Valid Cases

16-24

2.365

1.104

5.067

1.783

1.026

3.099

0.754

0.605

0.939

175

Page 4

**Odds Ratio**

95% Confidence Interval

Age groups (30 50)

Lower

Upper

ISS levels

Value

Odds Ratio for BAC 0 vs Any (0.00 / 1.00)

For cohort ICU admission = 0.00

For cohort ICU admission = ICU admission

N of Valid Cases

=>25

1.724

0.441

6.741

1.618

0.477

5.487

0.938

0.811

1.086

101

Odds Ratio for BAC 0 vs Any (0.00 / 1.00)

For cohort ICU admission = 0.00

For cohort ICU admission = ICU admission

N of Valid Cases

Total

1.687

1.286

2.213

1.255

1.107

1.422

0.744

0.641

0.862

1018

Odds Ratio for BAC 0 vs Any (0.00 / 1.00)

For cohort ICU admission = 0.00

For cohort ICU admission = ICU admission

N of Valid Cases

51-100

1-15

0.999

0.700

1.427

1.000

0.887

1.127

1.000

0.790

1.266

1149

Odds Ratio for BAC 0 vs Any (0.00 / 1.00)

For cohort ICU admission = 0.00

For cohort ICU admission = ICU admission

N of Valid Cases

16-24

1.079

0.480

2.425

1.058

0.580

1.927

0.980

0.795

1.209

274

Odds Ratio for BAC 0 vs Any (0.00 / 1.00)

For cohort ICU admission = 0.00

For cohort ICU admission = ICU admission

N of Valid Cases

=>25

0.922

0.240

3.539

0.931

0.289

3.007

1.010

0.850

1.201

130

Odds Ratio for BAC 0 vs Any (0.00 / 1.00)

For cohort ICU admission = 0.00

For cohort ICU admission = ICU admission

N of Valid Cases

Total

1.040

0.779

1.389

1.018

0.892

1.163

0.979

0.837

1.144

1553

Page 5

**Odds Ratio**

95% Confidence Interval

Age groups (30 50)

Lower

Upper

ISS levels

Value

Odds Ratio for BAC 0 vs Any (0.00 / 1.00)

For cohort ICU admission = 0.00

For cohort ICU admission = ICU admission

N of Valid Cases

Total

1-15

1.277

1.069

1.525

1.081

1.019

1.147

0.847

0.752

0.954

2918

Odds Ratio for BAC 0 vs Any (0.00 / 1.00)

For cohort ICU admission = 0.00

For cohort ICU admission = ICU admission

N of Valid Cases

16-24

1.401

0.933

2.104

1.259

0.945

1.677

0.899

0.796

1.015

624

Odds Ratio for BAC 0 vs Any (0.00 / 1.00)

For cohort ICU admission = 0.00

For cohort ICU admission = ICU admission

N of Valid Cases

=>25

1.044

0.554

1.967

1.038

0.603

1.787

0.994

0.908

1.087

368

Odds Ratio for BAC 0 vs Any (0.00 / 1.00)

For cohort ICU admission = 0.00

For cohort ICU admission = ICU admission

N of Valid Cases

Total

1.277

1.105

1.477

1.111

1.041

1.185

0.870

0.802

0.943

3910

**Mantel-Haenszel Common Odds Ratio Estimate**

Odds Ratio Ln(OR)

Standard Error of Ln(OR)

Asymptotic Significance (2-sided)

1.406

0.341

0.083

0.000

1.195

1.654

0.179

0.503

Asymptotic 95% Confidence Interval

Common Odds Ratio

Lower Bound Upper Bound Lower Bound

Upper Bound

Ln(Common Odds Ratio)

The Mantel-Haenszel common odds ratio estimate is asymptotically normally distributed under the common odds ratio of 1.000 assumption. So is the natural log of the estimate.

Page 6
