# Supplementary material for: Association of Blood Alcohol and Alcohol Use Disorders with Emergency Department Disposition of Trauma Patients
Source: West J Emerg Med. 2022 Feb 28;23(2):158–65. doi: 10.5811/westjem.2021.9.51376 (PMC8967454; doi:10.5811/westjem.2021.9.51376)
Supplement: Supplementary file 3 [file wjem-23-158-s003.docx]

| **Appendix 3: Association of Hospital admission with BAC level and age in patients with ISS: 1-15** | | | | | | | |
| --- | --- | --- | --- | --- | --- | --- | --- |
|  | | B | S.E. | Wald | df | P value | Odds Ratio |
|  | BAC Level | 0.15 | 0.01 | 175.87 | 1 | < 0.001 | 1.161 |
|  | age | 0.01 | 0.00 | 727.87 | 1 | < 0.001 | 1.012 |
|  | Constant | 2.43 | 0.03 | 7177.01 | 1 | < 0.001 | 11.408 |

| **Appendix 4: Association of Hospital admission with AUDIT score and age in patients with ISS: 1-15** | | | | | | | |
| --- | --- | --- | --- | --- | --- | --- | --- |
|  | | B | S.E. | Wald | df | P value | Odds Ratio |
|  | Audit categories | -0.09 | 0.01 | 63.58 | 1 | < 0.001 | 0.911 |
|  | age | 0.01 | < 0.001 | 234.40 | 1 | < 0.001 | 1.006 |
|  | Constant | 2.98 | 0.03 | 12355.56 | 1 | < 0.001 | 19.751 |

| **Appendix 5: Association of ICU admission with BAC level and age in patients with ISS: 1-15** | | | | | | | |
| --- | --- | --- | --- | --- | --- | --- | --- |
|  | | B | S.E. | Wald | df | P value | Odds Ratio |
|  | BAC Level | 0.05 | < 0.001 | 170.84 | 1 | < 0.001 | 1.056 |
|  | age | 0.01 | < 0.001 | 1081.35 | 1 | < 0.001 | 1.005 |
|  | Constant | -0.57 | 0.012 | 2310.11 | 1 | < 0.001 | 0.563 |

| **Appendix 6: Association of ICU admission with AUDIT score and age in patients with ISS: 1-15** | | | | | | | |
| --- | --- | --- | --- | --- | --- | --- | --- |
|  | | B | S.E. | Wald | df | P value | Odds Ratio |
|  | Audit categories | 0.23 | < 0.001 | 1765.81 | 1 | < 0.001 | 1.253 |
|  | age | 0.01 | < 0.001 | 1201.75 | 1 | < 0.001 | 1.005 |
|  | Constant | -0.76 | 0.012 | 4334.87 | 1 | < 0.001 | 0.466 |
